# Phylogenetic analysis of small ruminant lentiviruses in Germany and Iran suggests their expansion with domestic sheep

**DOI:** 10.1038/s41598-020-58990-9

**Published:** 2020-02-10

**Authors:** Vahid Molaee, Moira Bazzucchi, Gian Mario De Mia, Vahid Otarod, Darab Abdollahi, Sergio Rosati, Gesine Lühken

**Affiliations:** 1 Institute of Animal Breeding and Genetics, Justus Liebig University Giessen (JLU), Ludwigstraße 21, 35390 Gießen, Germany; 2 Istituto Zooprofilattico Sperimentale dell’Umbria e delle Marche Togo Rosati (IZSUM), Via G. Salvemini 1, 06126 Perugia, Italy; 3 Quarantine and Biosafety Directorate General, Iran Veterinary Organization (IVO), Vali Asr Avenue, Seyed Jamaledin Asad Abadi Street, 6349 Tehran, Iran; 4 Bureau of Animal Health and Disease Management, Iran Veterinary Organization (IVO), Vali Asr Avenue, Seyed Jamaledin Asad Abadi Street, 6349 Tehran, Iran; 5 Department of Veterinary Science, University of Turin (UNITO), Largo Paolo Braccini 2, 10095 Grugliasco Torino, Italy

**Keywords:** Coevolution, Palaeontology, Phylogenetics, Viral genetics, Retrovirus

## Abstract

Small ruminant lentiviruses (SRLVs) are found in sheep in Germany and Iran. SRLVs have been classified into four genotypes: A–C and E. Genotype A has been subdivided into 20 subtypes. Previous studies suggested that, first, the ancestors of genotype A are those SRLVs found in Turkey, second, the evolution of SRLVs is related to the domestication process, and, third, SRLV infection was first observed in sheep in Iceland and the source of that infection was a flock imported from Germany. This study generated, for the first time, partial SRLV sequence data from German and Iranian sheep, enhancing our knowledge of the genetic and evolutionary relationships of SRLVs, and their associations with the domestication process. Based on 54 SRLV sequences from German and Iranian sheep, our results reveal: (1) SRLV subtypes A4, A5, A11, A16 and A21 (new) are found in German sheep and A22 (new) in Iranian sheep. (2) Genotype A has potentially an additional ancestor (A22), found in Iran, Lebanon and Jordan. (3) Subtype A22 is likely an old version of SRLVs. (4) The transmission routes of some SRLVs are compatible with domestication pathways. (5) This study found no evidence of Icelandic subtype A1 in German sheep.

## Introduction

Small ruminant lentiviruses (SRLVs), which comprise maedi-visna virus (MVV) and caprine arthritis encephalitis virus (CAEV), belong to the genus Lentivirus and the family Retroviridae. SRLVs can cause progressive multisystem disease in sheep involving lungs, joints, mammary gland and the central nervous system^1^. There is no cure or vaccine available against SRLV infection. SRLV-related diseases are distributed worldwide among sheep and goats, resulting in considerable economic losses^2^.

Like in other lentiviruses, the SRLV genome includes three structural genes, coding for the group-specific antigens (*gag*), the polymerase (*pol*) and the envelope (*env*). The *gag* gene encodes the matrix (MA) protein (p17), capsid (CA) protein (p25) and nucleocapsid (NC) protein (p14)^3^. Both *gag* and *pol* genes are relatively conserved, and phylogenetic analyses of SRLVs have been established based on these two genes^4^.

SRLV isolates can be classified into four genotypes, A–C and E^4–6^. Genotypes A and B are widespread and refer to MVV-like and CAEV-like viruses, respectively. MVV-like and CAEV-like strains have been first described in sheep and goats, respectively, and considered strictly host-specific for a long time. However, there are nowadays several studies indicating that most strains can cross the species barrier (reviewed by Minardi da Cruz *et al*.^7^). Genotype A is the most heterogeneous group and has so far been subdivided into 20 subtypes, A1 to A20^4,8–15^. Two recently published studies have to be noted, one by Olech *et al*.^13^ that defines SRLV subtype A18, and the other by Colitti *et al*.^15^ that defines SRLV subtypes A18 and A19. In the present study, the SRLVs found by Colitti *et al*.^15^ are renamed from ‘A18’ to ‘A19’ and from ‘A19’ to ‘A20’. Genotype B contains three subtypes, B1 to B3^4,16^. SRLVs restricted to certain geographical areas have been assigned to other genotypes: genotype C is divided into two subtypes and refers to Norwegian isolates^15,17,18^, genotype D was found in few isolates originating from Switzerland and Spain, but they are now re-classified as genotype A^4,6,19^; genotype E comprises subtypes E1 and E2 and was isolated in Italy^9,20^.

Lentiviruses have a deep evolutionary history and have evolved alongside their mammalian hosts^21–25^. During the process of evolution, exogenous retroviruses (e.g. Jaagsiekte sheep retrovirus; a retrovirus that has many similarities with SRLVs and imposes pulmonary adenocarcinoma disease in sheep) have inserted into the germline of the infected host, leading to endogenous retroviruses (ERVs)^26^. In a study by Chessa *et al*.^27^, the presence of six variants of endogenous Jaagsiekte retrovirus (enJSRVs) was examined in 65 global domestic sheep, to investigate the history of sheep domestication. During the first wave of domestication, early domesticated sheep, which were morphologically wild but managed, appeared in the ancient Fertile Crescent region, including parts of Iran, Iraq, Turkey, Syria and Jordan, approximately 10,000 to 8,000 years before present (YBP)^28,29^. The domestication of goats started in the same region between 500 and 1,000 years earlier at about 11,000 YBP^28,30^. During the second wave of domestication, sheep with ‘modern’ features, typical of present-day breeds (e.g. with woolly fleeces and polled), appeared in West Asia and other areas in the world at approximately 6,000 YBP^27^. Host molecular genetic data combined with archaeological evidence indicates that sheep were distributed from the Fertile Crescent to the West and East likely during both waves of domestication^27,31,32^. For instance, during the first wave of domestication, the European mouflon migrated through the Mediterranean Basin to the islands of Corsica and Sardinia at around 7,000 YBP^21,27,32,33^. During the second wave, sheep with improved production traits were introduced from the East into Europe at the beginning of the 5th millennium YBP^34^.

In parallel with researches on host evolution, recent studies have demonstrated the usefulness of pathogens to elucidate the evolution of their hosts across time and location^21,35–37^. In this respect, besides enJSRVs^27^, investigating the phylogeny of SRLVs has the potential to enhance our knowledge of sheep and goat domestication^16,20,21^. The identification of SRLV subtype B3 in sheep/goats from Italy and Turkey as well as the finding that some bulk milk samples from Turkish sheep and goats were reactive against antigen derived from genotype E (a genotype found in Sardinia and other parts of Italy), supports the hypothesis of migration of domesticated sheep from the Fertile Crescent into the Mediterranean Basin during the Neolithic age^16^. However, phylogenetic studies involving SRLV sequences from other regions of the Fertile Crescent (except Turkey), which would potentially enhance our knowledge of the domestication process within the domestication origin itself, have been absent until now. While few SRLV sequences from Jordan and Lebanon are available in the database, no SRLV sequence information has been published from Iran, Iraq and Syria.

Historically, it has been suggested that maedi-visna was introduced to Iceland through German sheep^2,6,38–40^. In the early 1930s, a set of Karakul sheep (n = 20) was imported from Halle in Germany to Iceland. After several months, signs of maedi-visna were observed in some Icelandic sheep flocks, which hosted German Karakul rams^38^. The German Karakul flock originated from Astrakhan (Russia)^41^. The Icelandic SRLV strain (subtype A1) was first characterised by Sigurdsson *et al*.^38^ about 15 years after the observation of the first signs of maedi-visna in the Icelandic sheep flocks. Subtype A1 had already been detected in many countries^6^. However, German SRLVs have not yet been characterised.

In Iran, SRLV infection was first diagnosed in sheep using histopathological methods in 2001^42^. Following this first survey, SRLV infection was reported in different parts of Iran, using serological methods or PCR techniques^43–46^.

Currently, researchers have suggested three domestication pathways, from West Asia (Iran and Turkey) to Europe and Africa^32,47^. Germany is located on the end terminal of the Danubian pathway, Italy is located on the northern Mediterranean pathway and Morocco is located on the southern Mediterranean pathway^47^. In this respect, Iran belongs to the ancient Fertile Crescent region, where the initial domestication of sheep and goats occurred^28–30^. Also, the geographical position of Germany for investigating domestication events is important, as it has long been connected with the ancient Fertile Crescent via the Danube River^32,47^. This study aimed at generating, for the first time, SRLV sequence data from German and Iranian sheep, for which no such data has been available until now. The results were expected to enhance our knowledge of the genetic and evolutionary relationships of German and Iranian SRLVs, as compared to those from other countries, as well as their associations with the domestication process.

## Results

### SRLV sequences from German and Iranian sheep flocks

All DNA samples (n = 54), were successfully amplified using *gag-pol* primers. None of the sequences showed evidence of recombination. Information on *gag-pol* SRLV sequences collected from different sheep flocks in Germany and Iran, and the accession numbers are given in Table 1. Notably, due to unmatched sequence data for the alignment (Supplementary Fig. S1 online), genetic and phylogenetic analyses on *gag-pol* sequences correspond to a part of the *gag* gene. Based on initial analyses, 23 out of the 54 *gag* sequences were selected for phylogenetic and genetic analyses. Of the 23 selected sequences, 17 *gag* sequences belonged to 13 German sheep flocks and 6 corresponded to 6 Iranian sheep flocks.

**Table 1:** Information on *gag-pol* SRLV sequences collected from different sheep flocks in Germany and Iran.

sample no.	flock no.	country	German state/Iranian province	strain	accession number	proposed subtype
1	1	Germany	Baden Württemberg	BW1	MN233104	A16
2	2	Germany	Bayern	BY1	MN233105	A5
3	3	Germany	Hessen	HE1	MN233106	A21
4	3	Germany	Hessen	HE2	MN233107	A5
5	4	Germany	Nordrhein-Westfalen	NW1	MN233108	A5
6	5	Germany	Nordrhein-Westfalen	NW2	MN233109	
7	5	Germany	Nordrhein-Westfalen	NW3	MN233110	
8	5	Germany	Nordrhein-Westfalen	NW4	MN233111	A21
9	5	Germany	Nordrhein-Westfalen	NW5	MN233112	
10	5	Germany	Nordrhein-Westfalen	NW6	MN233113	
11	6	Germany	Schleswig-Holstein	SH1	MN233114	
12	6	Germany	Schleswig-Holstein	SH2	MN233115	
13	6	Germany	Schleswig-Holstein	SH3	MN233116	A21
14	7	Germany	Schleswig-Holstein	SH4	MN233117	
15	7	Germany	Schleswig-Holstein	SH5	MN233118	A21
16	8	Germany	Schleswig-Holstein	SH6	MN233119	
17	8	Germany	Schleswig-Holstein	SH7	MN233120	A21
18	8	Germany	Schleswig-Holstein	SH8	MN233121	
19	8	Germany	Schleswig-Holstein	SH9	MN233122	
20	8	Germany	Schleswig-Holstein	SH10	MN233123	
21	8	Germany	Schleswig-Holstein	SH11	MN233124	A11
22	8	Germany	Schleswig-Holstein	SH12	MN233125	
23	8	Germany	Schleswig-Holstein	SH13	MN233126	
24	9	Germany	Schleswig-Holstein	SH14	MN233127	
25	9	Germany	Schleswig-Holstein	SH15	MN233128	A21
26	9	Germany	Schleswig-Holstein	SH16	MN233129	
27	9	Germany	Schleswig-Holstein	SH17	MN233130	
28	9	Germany	Schleswig-Holstein	SH18	MN233131	
29	9	Germany	Schleswig-Holstein	SH19	MN233132	A21
30	9	Germany	Schleswig-Holstein	SH20	MN233133	
31	9	Germany	Schleswig-Holstein	SH21	MN233134	
32	9	Germany	Schleswig-Holstein	SH22	MN233135	
33	9	Germany	Schleswig-Holstein	SH23	MN233136	
34	10	Germany	Schleswig-Holstein	SH24	MN233137	
35	10	Germany	Schleswig-Holstein	SH25	MN233138	
36	10	Germany	Schleswig-Holstein	SH26	MN233139	
37	10	Germany	Schleswig-Holstein	SH27	MN233140	
38	10	Germany	Schleswig-Holstein	SH28	MN233141	A21
39	10	Germany	Schleswig-Holstein	SH29	MN233142	
40	10	Germany	Schleswig-Holstein	SH30	MN233143	A11
41	11	Germany	Schleswig-Holstein	SH31	MN233144	
42	11	Germany	Schleswig-Holstein	SH32	MN233145	A21
43	11	Germany	Schleswig-Holstein	SH33	MN233146	
44	11	Germany	Schleswig-Holstein	SH34	MN233147	
45	12	Germany	Schleswig-Holstein	SH35	MN233148	A4
46	13	Germany	Schleswig-Holstein	SH36	MN233149	
47	13	Germany	Schleswig-Holstein	SH37	MN233150	
48	13	Germany	Schleswig-Holstein	SH38	MN233151	A11
49	14	Iran	Chaharmahal-Va-Bakhtiari	BKH1	MK098477	A22
50	15	Iran	Chaharmahal-Va-Bakhtiari	BKH2	MK098478	A22
51	16	Iran	Kerman	KRM1	MK098479	A22
52	17	Iran	Kerman	KRM2	MK098480	A22
53	18	Iran	Western Azarbaijan	MKU1	MK098481	A22
54	19	Iran	Western Azarbaijan	MKU2	MK098482	A22

### Phylogenetic analysis of SRLVs based on *gag* fragment

**Figure 1:**
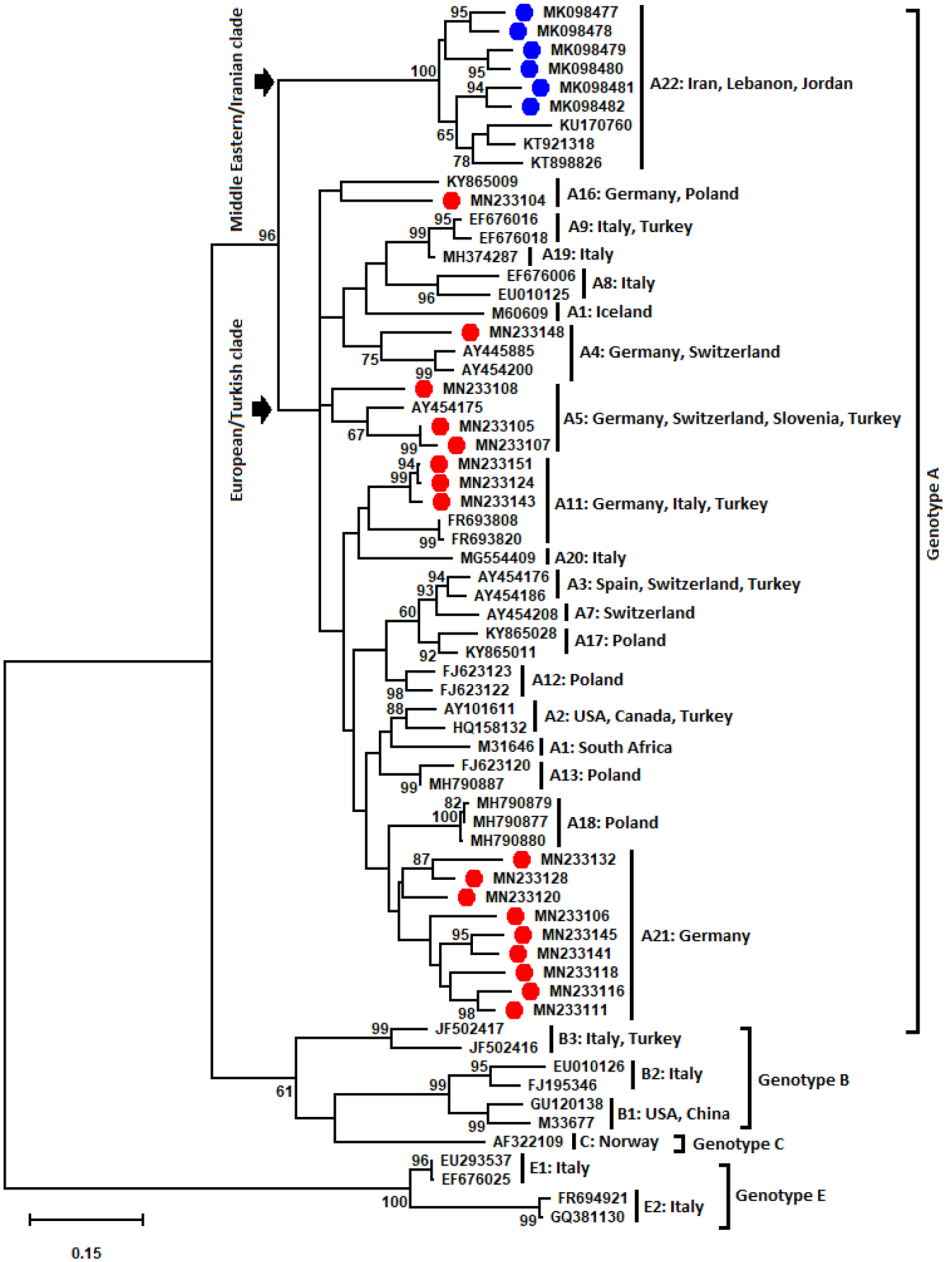
Phylogenetic tree indicates SRLV subtypes and their differing geographical distribution (country). The analysis was performed using the Maximum Likelihood method and was based on the Tamura-Nei model^61^. The analysis involved a total of 399 bp from 65 nucleotide sequences: 17 German *gag* sequences (labelled by solid red circles), six Iranian sequences (labelled by solid blue circles), two Jordanian SRLV sequences (KT898826 and KT921318), a Lebanese SRLV sequence (KU170760) and 39 reference SRLV sequences originating from different geographical areas (retrieved from the GenBank database). The position of the analysed fragments was related to coding regions of the capsid gene (p25) and the nucleocapsid gene (p14) located on the *gag* fragment (nucleotide: 1114–1506; numbering according to prototype strain K1514^62^. The difference in the evolutionary rate among sites was considered using the discrete gamma distribution ( + G parameter = 0.5202) and the invariable sites ( + I = 21.68% sites). The numbers on the nodes indicate the percentage of bootstrap values obtained from 1,000 replicates. Bootstrap values of less than 60% were excluded from nodes. The branch lengths show the number of substitutions per site. Evolutionary analyses were performed in MEGA7^60^. The two suggestive clades of genotype A, “European/Turkish clade” and “Middle Eastern/Iranian clade” are shown with arrows.

The constructed phylogenetic tree with *gag* sequences is shown in Fig. 1. According to the phylogenetic analysis, the *gag* sequences from Germany (n = 17), which were distributed in five distinct clusters, were strongly related to genotype A. The tree showed that these sequences were affiliated to subtypes A4 (flock 12), A5 (flocks 2–4), A11 (flocks 8, 10 and 13), A16 (flock 1) and potentially to a new subtype, which could be tentatively named A21 (flocks 3 and 5–11). Not all of these clusters were supported with high bootstrap values. The six Iranian SRLV sequences belonged to genotype A with a bootstrap value of 96%. Furthermore, the Iranian SRLV sequences clustered together with the Jordanian (KT898826 and KT921318) and Lebanese (KU170760) SRLV prototypes. This cluster was tentatively named A22.

### Genetic distance analyses of German and Iranian SRLV sequences based on *gag* gene

As all German and Iranian *gag* sequences tend to be genotype A, a cut-off value was determined for assigning a new subtype within genotype A. The mean genetic distances between available *gag* reference data (A1–A20 except for A6, A10, A14 and A15) was 14.67% (SE = 0.19%, 95%CI = 14.29–15.05%). The value of 14.67% was the used cut-off value for defining a new subtype within genotype A. German *gag* sequences of the proposed subtype A21 (n = 9), had mean sequence similarity of 11.62% (range = 0.06–15.01%). The following mean genetic distances between the new subtype (A21) and different genotypes were observed: A: 15.15%, range = 13.94–16.81%; B: 23.11%, range = 22.01–24.17%; C: 23.61%, range = 21.88–25.95%; E: 28.50%, range = 27.42–29.46% (Table 2 and Supplementary Table S1 online). Based on phylogeny, the most similar subtype with cluster A21 was A18. In two out of nine *gag* sequences of cluster A21 (MN233132 and MN132145), genetic distances compared to A18 were higher than the cut-off value. Observation of these two sequences in cluster A21 that genetically differed from A18 (>14.67), fulfils the criterion of proposing a new subtype for a cluster in SRLVs^4^. Thus, all nine sequences of cluster A21 constitute subtype A21 (new). In other German *gag* sequences, the genetic distances with the most similar subtypes (corresponding to subtypes A4, A5, A11 and A16 based on phylogeny) were lower than the cut-off value (<14.67). Therefore, other German *gag* sequences did not constitute new subtypes and joined the reference subtypes A4 (MN233148), A5 (MN233105, MN233107 and MN233108), A11 (MN233124, MN233143 and MN233151) and A16 (MN233104). Notably, according to phylogeny (Fig. 1), the most similar subtypes with the three sequences of MN233143, MN233124 and MN233151 were A11 and A20 (both SRLV subtypes from Italy). Based on genetic distance analysis these three sequences were more similar with A11 than with A20. More details are shown in Table 2 and Supplementary Table S1 online.

**Table 2:** Estimates of evolutionary divergences of German and Iranian SRLV sequences compared to different subtypes of genotype A based on the *gag* fragment (nucleotide: 1114–1506; numbering according to prototype strain K1514, Staskus *et al*.^62^).

sequence	country	A1	A2	A3	A4	A5	A7	A8	A9	A11	A12	A13	A16	A17	A18	A19	A20	Mean A
MN233148	Germany	17.43	16.28	14.89	13.23	12.98	15.27	16.54	16.69	16.79	15.78	15.39	17.05	14.41	16.92	14.50	17.30	15.72
MN233105	Germany	16.41	15.01	13.23	16.03	9.41	13.49	17.18	13.50	15.78	13.61	13.61	16.28	11.35	14.12	12.21	13.74	14.06
MN233108	Germany	16.16	14.50	14.89	15.65	12.21	14.50	18.07	15.92	13.23	14.89	14.63	15.01	15.69	14.08	16.03	15.27	15.05
MN233107	Germany	16.28	15.52	12.47	16.79	9.92	12.72	17.94	13.76	15.78	13.61	13.10	16.79	11.10	13.10	12.47	14.76	14.13
MN233143	Germany	14.50	11.96	14.12	16.92	14.25	15.52	16.92	15.29	12.98	12.98	13.49	17.56	14.16	12.47	14.76	12.98	14.43
MN233124	Germany	14.38	11.20	14.25	16.92	13.99	15.52	17.05	14.27	12.47	12.21	13.49	16.03	13.90	12.72	14.25	13.49	14.13
MN233151	Germany	14.38	10.69	14.25	16.41	13.49	15.52	17.05	14.27	12.47	12.21	13.49	16.03	13.39	12.72	13.74	13.49	13.98
MN233104	Germany	15.78	16.28	17.18	16.54	16.03	16.79	19.47	18.73	17.18	15.14	15.27	14.25	14.29	15.52	17.30	15.78	16.35
MN233116	Germany	16.67	15.52	15.39	18.70	15.78	15.27	18.83	16.82	14.50	14.63	14.89	17.56	15.43	14.63	16.54	16.28	16.09
MN233111	Germany	15.52	13.87	14.12	19.85	13.74	15.01	18.07	15.80	13.49	13.87	13.10	15.78	14.80	14.25	13.99	15.52	15.05
MN233118	Germany	15.65	13.49	14.89	17.94	13.99	15.01	17.94	15.54	15.52	13.74	12.98	17.05	13.78	13.10	15.01	16.79	15.15
MN233132	Germany	18.32	15.14	16.16	17.43	15.52	16.54	20.48	18.47	15.65	15.27	15.52	18.32	15.94	16.03	16.79	17.30	16.81
MN233120	Germany	14.76	12.47	15.01	15.78	14.25	14.50	16.92	15.41	12.85	12.47	10.81	14.50	13.65	11.70	14.25	13.74	13.94
MN233145	Germany	16.16	14.38	11.83	16.79	14.50	11.96	16.92	15.16	13.49	11.07	14.38	16.28	13.90	16.28	14.76	16.28	14.63
MN233128	Germany	16.67	12.98	14.38	15.39	13.23	14.50	18.32	16.94	16.16	13.10	11.45	16.28	14.41	11.79	14.25	17.05	14.80
MN233141	Germany	16.92	13.74	13.49	17.18	13.99	13.23	18.58	16.31	14.76	14.50	14.50	17.30	13.78	13.74	15.27	15.27	15.16
MN233106	Germany	15.14	13.61	13.99	16.79	13.99	14.76	16.28	14.90	15.01	14.76	14.12	16.28	12.76	12.60	13.74	16.28	14.69
MK098477	Iran	19.34	18.32	17.81	18.32	15.01	17.81	19.72	19.24	19.59	18.07	19.21	18.83	16.58	18.15	18.32	18.83	18.32
MK098478	Iran	19.97	18.58	18.70	18.96	15.78	19.08	20.48	19.49	18.19	17.68	20.23	20.10	18.11	18.24	19.08	19.59	18.89
MK098479	Iran	20.10	18.07	15.65	19.72	15.27	17.05	18.58	16.69	18.07	17.30	20.23	19.08	17.47	19.93	16.28	16.54	17.88
MK098480	Iran	19.85	19.21	15.65	19.59	15.78	16.28	17.18	16.82	18.83	17.94	19.59	19.85	16.45	19.17	16.79	16.54	17.85
MK098481	Iran	19.21	19.59	17.94	19.21	16.03	19.34	20.74	18.85	17.94	17.05	18.96	19.34	18.37	18.83	18.32	17.30	18.56
MK098482	Iran	18.58	18.96	16.92	19.47	15.01	18.58	19.34	17.32	17.81	17.30	19.08	19.85	17.35	19.76	18.07	18.32	18.23

For each reference subtype, up to three strains were selected and the evolutionary divergences calculated based on the mean divergence of each set of subtypes and representative sequences of this study. 17 German and six Iranian SRLV sequences were compared with subtypes A1 (M60609 and M31646), A2 (AY101611 and HQ158132), A3 (AY454186 and AY454176), A4 (AY454200 and AY445885), A5 (AY454175), A7 (AY454208), A8 (EU010125 and EF676006), A9 (EF676018 and EF676016), A11 (FR693820 and FR693808), A12 (FJ623122 and FJ623123), A13 (MH790877and FJ623120), A16 (KY865009), A17 (KY865011 and KY865028), A18 (MH790877, MH790879 and MH790880), A19 (MH374287) and A20 (MG554409).

The *gag* sequences of six Iranian sheep constituted a cluster (subtype A22) within genotype A. The mean genetic similarity between Iranian SRLVs was 10.62% and varied from 5.34% to 12.72%. The following mean genetic distances between Iranian SRLV sequences (A22) and different genotypes were observed: A: 18.29%, range = 17.85–18.89%; B: 23.45%, range = 22.31–24.77%; C: 24.13%, range = 22.65–25.45%; E: 28.73%, range = 27.87–29.84% (Table 2 and Supplementary Table S1 online). The genetic divergences between six Iranian, two Jordanian (KT898826 and KT921318) and one Lebanese (KU170760) *gag* sequences were lower than the cut-off value (<14.67). Therefore, all these nine *gag* sequences together constitute subtype A22. More details are shown in Table 2 and Supplementary Table S1 online.

### Amino acid (aa) sequence analysis based on the *gag-pol* fragment

**Figure 2:**
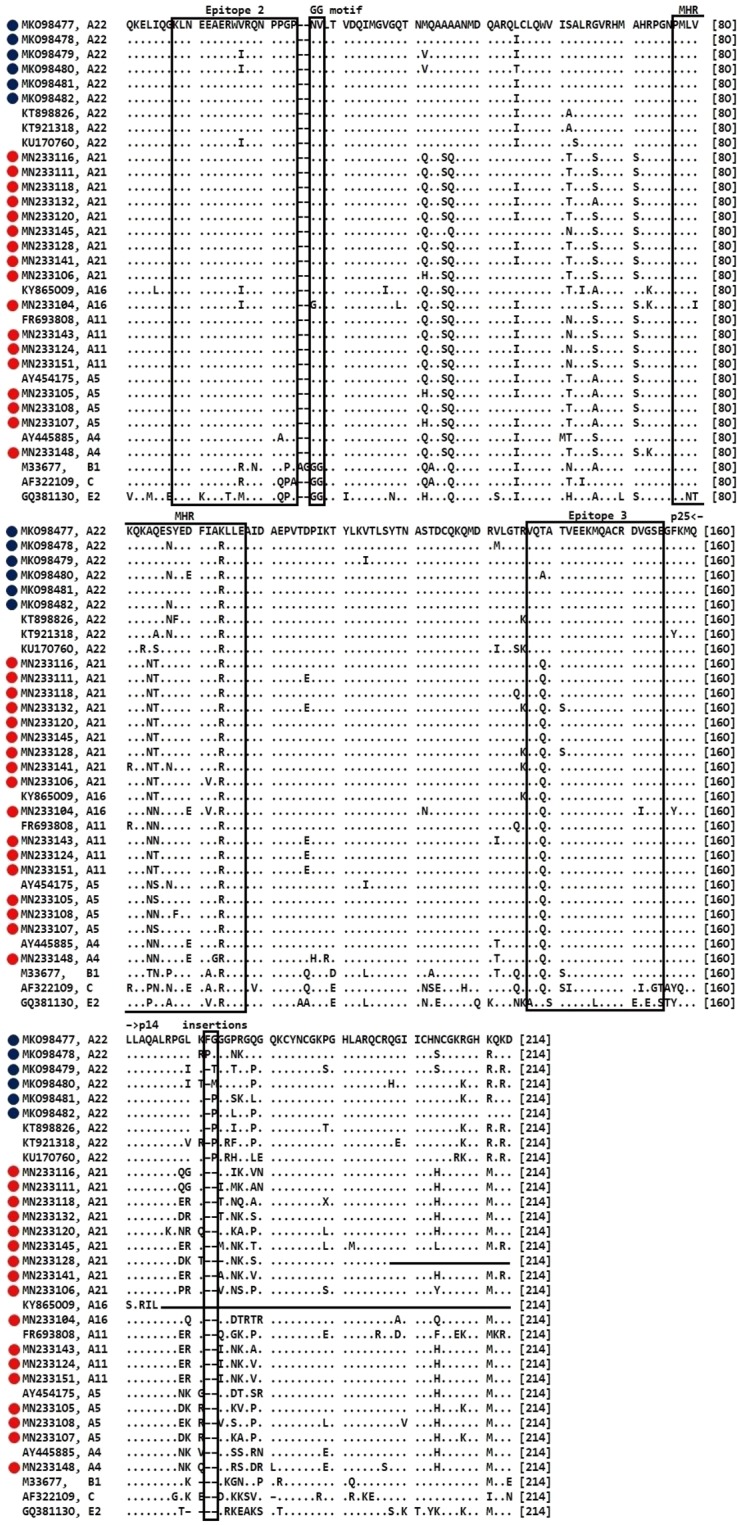
Amino acid sequence alignments of the SRLV *gag-pol* fragment. The reference sequence is the SRLV sequence of strain BKH1 (accession number: MK098477) from the Iranian province of Chaharmahal-Va-Bakhtiari. For this amino acid comparison, 6 Iranian (labelled by solid blue circles) and 17 German sequences (labelled by solid red circles) were compared with different SRLV subtypes/genotypes. Immunodominant epitopes 2 and 3, the major homology region (MHR), the double glycine motif (GG) and insertions (at position 172 or 173) are delineated with boxes. The major core protein (p25) and nucleic acid-binding protein (p14) are separated with left and right arrows (p25 ≤ position 160; p14 ≥ position 161). Dashes and dots indicate deletions and identical residues, respectively.

The alignment of 65 *gag-pol* aa sequences (up to 214 aa) is shown in Fig. 2 and Supplementary Fig. S2 online. The reference *gag-pol* sequence in both Fig. 2 and Supplementary Fig. S2 online is the Iranian SRLV strain of BKH1 (accession number: MK098477). Except for the reference *gag-pol* sequence (MK098477), the *gag-pol* aa sequences in Fig. 2 (n = 32) and Supplementary Fig. S2 online (n = 32) are not identical. Therefore, the sum of *gag-pol* aa sequences that have been aligned with MK098477 is 64, and altogether a total of 65 *gag-pol* aa sequences is shown in Fig. 2 and Supplementary Fig. S2 online.

Both German and Iranian SRLV sequences share similar epitope patterns with other sequences of genotype A. Also, they did not share the double glycine “GG” motif^48^, as other sequences of genotype A (Fig. 2). When comparing amino acid sequence substitutions at epitopes 2, major homology region (MHR) and epitopes 3, few alterations were observed mostly in the middle part of the conserved SRLV domains found in Germany and Iran.

All the Iranian SRLVs, as well as the Jordanian (KT898826 and KT921318) and Lebanese (KU170760) strains, contained an insertion at position 173, which was not seen in any other small ruminant lentiviruses (Fig. 2 and Supplementary Fig. S2 online). A second insertion at position 172 was found exclusively in two SRLV *gag-pol* sequences in sheep flocks from the Iranian province of Chaharmahal-Va-Bakhtiari. Interestingly, these insertions (positions 172 or 173) are found exclusively in SRLV subtype A22 and as well in other lentiviruses, including bovine immunodeficiency virus (BIV), human immunodeficiency virus type 1 (HIV1), simian immunodeficiency virus (SIV), feline immunodeficiency virus (FIV), equine infectious anemia virus (EIAV) and human immunodeficiency virus type 2 (HIV2) (Supplementary Fig. S3 online).

### Geographical distribution of SRLV subtypes found in this study

**Figure 3:**
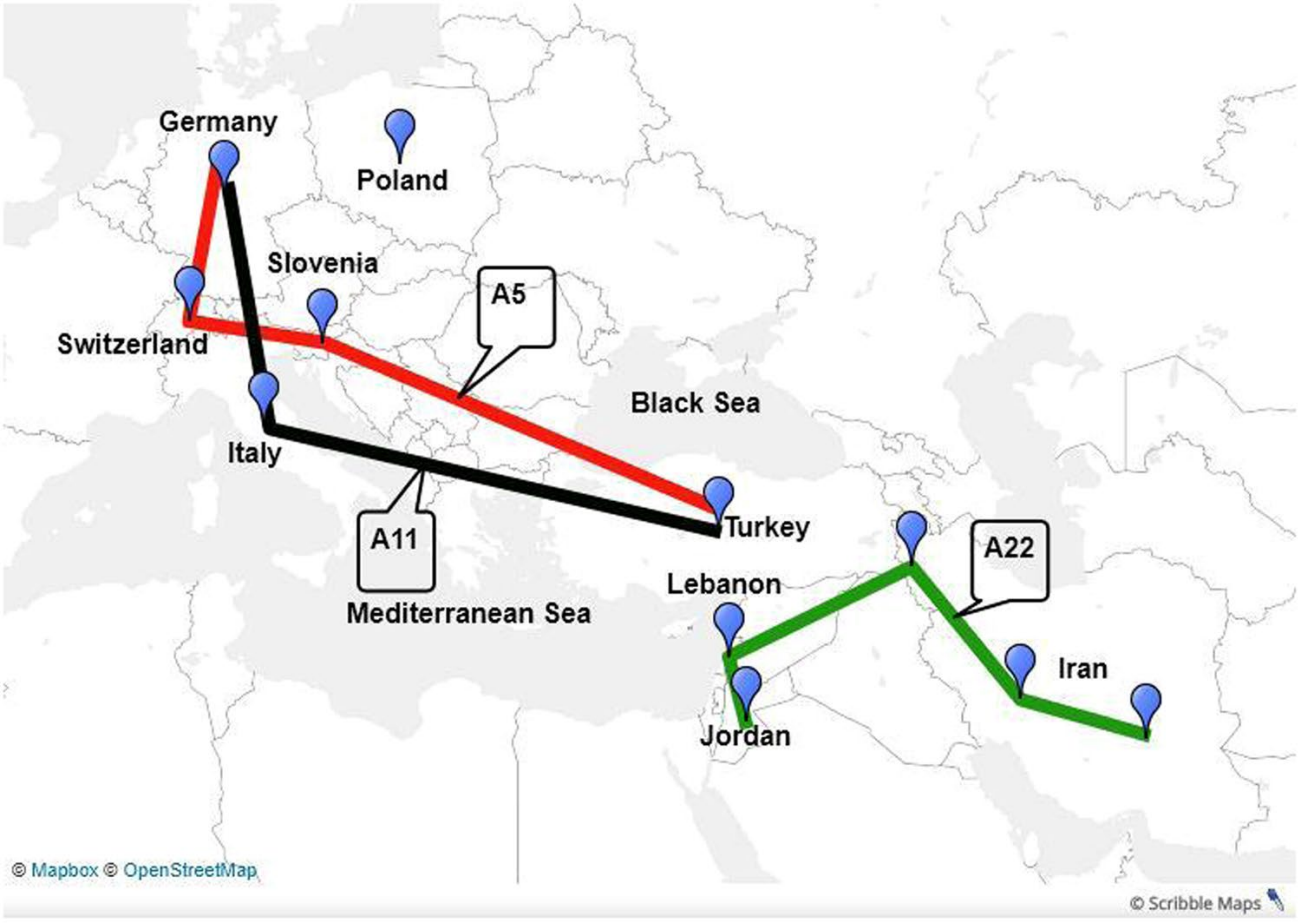
Putative transmission routes of SRLV subtypes A5, A11 and A22 based on their geographical distribution. The Danubian domestication pathway, corresponding to the Danube River, rises in Germany, flows through different European countries, and finally reaches the Black Sea. The northern Mediterranean pathway comprises Turkey and southern parts of Europe, including Italy. The ancient Fertile Crescent region incorporates Iran, Iraq, Turkey, Syria, Lebanon and Jordan (based on Harlan and Zohary^57^). The map was created using online Scribble Maps (https://www.scribblemaps.com/). For more information concerning printing permissions and inclusion of logos please visit https://help.scribblemaps.com/knowledgebase/articles/878916-printing-permissions-book-offline-etc. The online Scribble Maps used the databases of “© OpenStreetMap”, “© Mapbox” and “Improve this map”. The OpenStreetMap provides free geographic data and is available under the Open Database Licence (https://www.openstreetmap.org/copyright). To learn more about the Mapbox, visit https://www.mapbox.com/about/maps/. Further information regarding the use of “Improve this map” is available at https://www.mapbox.com/map-feedback/. The URL for linking to the map in Fig. 3 is https://www.scribblemaps.com/maps/view/Figure_3_/XUxAbALsVL.

SRLV subtypes of A4, A5, A11, A16, A21 and A22 were observed in this study. Except for A21, the other SRLVs have also been found in other countries. The qualitative aspects of the phylogeography were presented in Fig. 3 by showing the geographic dispersal of pairwise SRLV subtypes of A5, A11 and A22. Transmission routes of SRLV subtypes A5 (solid red line), A11 (solid black line) and A22 (solid green line) follow the Danubian pathway, northern Mediterranean pathway and the ancient Fertile Crescent, respectively. As subtype A4 was only determined in Germany and Switzerland^4^, and subtype A16 was only observed in Germany and Poland^12^, for simplicity, their transmission routes were not shown in Fig. 3.

## Discussion

Maedi-visna disease, already distributed among the sheep flocks of Germany^41,49^, was first detected in the German East (1967) and then in the West (1972)^41^. It was reported in southwestern Iran in 2001^42^ and later in other parts of the country^43–45^. In this study, for the first time, *gag* sequences of German and Iranian SRLVs were generated and analysed, using samples from five German states and three Iranian provinces. Our analyses identified a noticeable variation in German SRLV sequences that was absent in the Iranian ones. In the 13 studied German flocks, SRLV subtypes A4, A5, A11, A16 and A21 were found, with co-infection being identified in three flocks: flock 3 (A5 and A21), flocks 8 and 10 (A11 and A21) (Table 1). In the six Iranian flocks, only subtype A22 was found.

In earlier studies, migration of at least some SRLVs was related to the old times^16,50^. For example, subtype B3 and genotype E, which are rare, were found in Turkey and Italy (Sardinia and other regions)^16^. Turkey is one of the centres of sheep and goat domestication^28,32^, and the early agriculture of Sardinia is related to the Neolithic period^21,27,32,33^. Later, observations of various SRLV subtypes of genotype A (A2, A3, A5, A9 and A11) in sheep flocks of Turkey let Muz *et al*.^50^ propose that the ancestors of all SRLVs, especially genotype A, are Turkish SRLVs, and the evolution of some SRLVs is related to the domestication process or a more recent transmission pathway during the Ottoman Empire (about 14th–19th centuries). Our results are consistent with this scenario, which relates the evolution of SRLVs to the domestication process^16,50^.

So far, SRLV subtype A22 has been observed in sheep in Iran, Lebanon and Jordan but not in any European countries, including Turkey. The absence of A22 among small ruminants of Turkey and Europe is likely related to their hosts’ lineages. Several reports have shown that domestic sheep have different wild ancestors. Already, five lineages (A–E) have been identified in modern domestic sheep through global studies of mitochondrial DNA^51–55^. There is a relatively low percentage of lineages C–E, whilst the two most frequent lineages in domestic sheep are A and B, as identified by Hiendleder and colleagues^51,52,55^. These two lineages could be linked, but not completely, with modern fat- and thin-tailed where the first one is mostly found in Iran and Eastern Asia and the other one in Europe (including Turkey)^56^. Possibly, this could be a result of geographical separation of sheep lineages in the past. Accordingly, a long-term genetic differentiation took place between SRLV subtype A22, found in Iran, Lebanon and Jordan and other SRLVs of genotype A, found in Europe. Therefore, A22 could be suggested as an additional ancestor for genotype A of SRLVs (see Fig. 1; European/Turkish clade vs. Middle Eastern/Iranian clade).

In this study, we realised that the relationship between transmission routes of some SRLVs (A5, A11 and A22, this study; A3, A9, B3 and E reviewed by Ramírez *et al*.^6^) are compatible with domestication pathways of sheep. Three domestication pathways have been proposed from the Near East (Iran and Turkey) to the West and Africa^32,47^. The first pathway follows the Danube River from the Near East (Turkey) to Germany^32,47^. An excellent example of this distribution is the observation of SRLV subtype A5 in Turkey^50^ (start terminal), Slovenia^10^, Switzerland^4^ and Germany (end terminal). This subtype’s existence in countries lying on the route between Turkey and Germany (Fig. 3, red line) shows that the distribution is targeted. A further hint for the existence of this pathway is SRLV subtype A3, which has been identified in Turkey, Switzerland and Spain (reviewed by Ramírez *et al*.^6^). The second domestication pathway runs from the Near East via the Mediterranean route to southern Europe^32,47^, including Italy. SRLV subtype A11 hints at this distribution. It has been detected in Turkey^50^ and Italy^8^ and in the current study also in Germany (Fig. 3, black line). Other clues for the second domestication pathway could be related to the previously observed subtypes A9, B3 and genotype E in Turkey and Italy^8,16,50^. The third proposed domestication pathway runs from the Near East via the southern Mediterranean route to the northern parts of Africa^32,47^. A good support of this distribution is shown by the observations of A22 in Iran, Lebanon and Jordan; these observations are also compatible with the location of the ancient Fertile Crescent^57^ (Fig. 3, green line), where domestication originated. Therefore, our data reflect an association between the distribution of domestication and the transmission of some SRLVs. However, the distribution of some SRLV subtypes may not be related to antiquity. For example, subtype A16 was found only in Germany and Poland^12^. Subtype A21, the most common subtype in the North of Germany, is genetically close to subtype A18^13^ in Poland. A study by Olech *et al*.^13^ mentioned an epidemiological linkage between the small ruminants of Germany and Poland after the Second World War, which is supported by our results.

Amino acid insertions 172 or 173 were observed in SRLV subtype A22 but were not found in any other subtypes. Notably, we observed these insertions to be typical for subtype A22 and other lentiviruses but not for all other SRLVs. As these insertions are in a variable area of the *gag-pol* region (Supplementary Fig. S3 online), bioinformatics analyses may not show how these insertions have evolved in different lentiviruses. Observation of these insertions, however, confirms evolutionary processes within and between different lentiviruses. Furthermore, the hosts of the Jordanian isolates were sheep of the Awassi breed (M. Mazzei, University of Pisa, Italy, personal communication), which is a fat-tailed sheep with a history related to the second wave of domestication (about 5,000 years ago)^58^. Likewise, the Iranian samples came from three provinces close to the Zagros Mountains that are also known as primary centres of goat and sheep domestication^29,30^. The common presence of a unique variant of SRLVs (insertion at positions 172 or 173) among sheep from the countries of the ancient Fertile Crescent suggests that subtype A22 may represent an ancient precursor of modern SRLVs.

The detection of a single SRLV A subtype in Iranian, Jordanian and Lebanese sheep also suggests that these sheep have lived in isolation for centuries. The restricted variability points to this SRLV subtype as a potential marker to trace sheep domestication pathways. In contrast, the situation in Europe may be much more complex and the influence of trading overwhelming, confounding the potential link of SRLV genotypes to the domestication pathways, such as the Danubian or northern Mediterranean pathways. In this respect, Chessa and colleagues^27^ showed that tracking infection and endogenization of the Jaagsiekte sheep retrovirus in different sheep breeds permits the reconstruction of the domestication history of sheep. Unlike SRLVs, Jaagsiekte sheep retrovirus introduces a stable marker in the genome of the infected animals and may be more suitable to follow the domestication pathways of the species under study. Therefore, further studies on sheep genome variations using endogenous retroviruses may provide a more precise picture of the domestication pathways in Europe.

Earlier reports mentioned that maedi-visna (subtype A1) arrived in Iceland with Karakul sheep imported from Germany. Straub^41^ showed that this Karakul flock existed in Germany until 1970 but that the sheep of this flock had never shown any signs of maedi-visna. Currently, there is a meager number of Karakul sheep in Germany (maybe a single breeding flock), and this flock is not known to be SRLV positive. However, SRLVs are not strictly breed-specific. They can circulate from one region to another and as well from one breed to another. In this study, we found no evidence for the presence of the Icelandic subtype (A1) among 48 *gag* sequences in German sheep. However, it is possible that there are additional subtypes or genotypes of SRLVs present in German sheep flocks that have not yet been identified, including A1. More work is therefore needed to firmly rule out the hypothesis of distribution of subtype A1 via Karakul sheep from Germany to Iceland.

Shah *et al*.^4^ developed a systematic taxonomic classification for SRLVs, based on 1.8-kb *gag-pol* sequences. At that time and later, many strains have been included in the list of new SRLVs when the characteristic of a new strain was distinct enough from other strains. However, in different research projects, different fragments of *gag-pol* were sequenced. The available sequence data for the SRLV subtypes A12, A13, A14, A16, A17 and A18 were not fully matched to the sequence data of other SRLV subtypes of genotype A. As a result, the alignment and classification of SRLVs were limited to only 0.4-kb of *gag* gene (Supplementary Fig. S1 online). In this study, we used the same analysis for defining the cut-off value as it was used by Shah *et al*.^4^. As the 0.4-kb *gag* fragment we used in this study is more conserved than the 1.8-kb *gag-pol* fragment used by Shah *et al*.^4^, we adapted the cut-off value (14.67% instead 15%) in order to get an accurate classification.

The principal limitation of this study is the restricted number of sequences obtained. 48 samples were collected in Germany, 38 of which came from Schleswig-Holstein, 6 from Nordrhein-Westfalen, and the remaining 4 from Baden-Württemberg, Hessen and Bayern. It is highly unlikely that these sequences are representatives of the SRLV strains circulating in this country. The same applies to Iran, with only six sequences. The reason for unbalanced sequencing results from German flocks is the identification of more positive flocks in the North of Germany^49^. Although Germany is not free of SRLVs, there are not many infected flocks, especially of low susceptible breeds^46^. German Texel is a sheep breed susceptible to SRLV infection and it is mostly found in the state of Schleswig-Holstein (North of Germany)^49^. We found less/no positive flocks in other German states. The six Iranian SRLV sequences were collected in a previous study by collecting samples from 30 sheep flocks in six Iranian provinces. We found SRLV positive flocks only in three provinces of Western Azarbaijan, Kerman and Chaharmahal-Va-Bakhtiari^46^. Similar to the situation in Germany, the distribution of SRLV positive samples in these three Iranian provinces was not balanced. Finally, we decided to select two samples per each province. Therefore, performing follow up studies on characterisation of SRLVs in sheep flocks from additional regions in Germany and Iran is necessary to provide a more in-depth insight into the variability of SRLVs circulating in both countries.

## Materials and Methods

### Samples

For characterisation of German and Iranian SRLVs, a total of 54 DNA samples was selected from SRLV positive sheep flocks in Germany (13 flocks, n = 48 samples) and Iran (6 flocks, n = 6 samples)^49^. The SRLV infection status of German and Iranian sheep flocks were determined in earlier studies based on serological (IDEXX CAEV-MVV Total Ab ELISA, Ludwigsburg, Germany)^49^ and PCR (a semi-nested PCR on *env* gene)^46^ tests, respectively. Origin of flocks and the number of sequenced samples from each flock in this study are shown in Table 1.

### Ethical approval

No live animals were used for this study. All blood samples were collected by trained veterinarians exclusively as part of a routine clinical examination and the residuals of the samples would have otherwise been discarded. According to the German regulations for animal protection, the German Animal Welfare Act (released on 8/5/2006, modified on 17/12/ 2018), this origin of samples obviates the need for an explicit ethics committee approval. Iran Veterinary Organization (IVO), a national authority, participated in collecting Iranian sheep samples (permission issued on 20/12/2014, No: 93/22/70521). The ethical responsibility for Iranian animal welfare was carried out in accordance with the legal regulations of the IVO.

### PCR amplification and sequencing of SRLV proviral DNA

To characterise the SRLVs found in Germany and Iran, genomic DNA of sampled sheep was analysed using a nested PCR targeting the *gag-pol* region of the provirus as described and utilised elsewhere^8,9,50^.

For sequencing purposes, PCR products of the second PCR were purified and directly sequenced with either ABI PRISM 3130 Genetic Analyzer or by LGC Genomics GmbH, Germany (https://shop.lgcgenomics.com). To control PCR errors, both the sense and antisense strands were sequenced performing three independent reactions for each sample.

### Analyses of SRLV sequence data

The obtained SRLV provirus nucleotide sequences were trimmed and analysed using the ChromasPro software version 2.1.6 (Technelysium Pty Ltd, Tewantin, Australia). Recombination analyses were carried out using RDP4 package^59^. Multiple alignments of the nucleotide sequences and the deduced amino acid sequences were generated with Muscle-built in Mega version 7.0.26^60^.

For simplicity, only one or two sequences (Table 1) of each flock were selected and used for all analyses. The available sequences of the reference SRLV strains of genotypes A–C and E, and the SRLV strains most homologues to German and Iranian SRLVs (using the Basic Local Alignment Search Tool, BLAST) were included in the analysis. The sequence data of SRLV subtypes A6, A10, A15 were not available (a pol fragment defined these subtypes) for the analyses. Additionally, A14 had to be excluded from analyses because of the shortness of the relevant sequence part. All analyses were carried out based on 65 aligned sequences from various sources (Germany 17, Iran 6, Jordan 2, Lebanon 1 and 39 from reference SRLV subtypes).

The evolutionary relationships of German and Iranian SRLVs with other published sequences were investigated by constructing the phylogenetic tree from alignments of the *gag* region. The best-fitting nucleotide substitution model for phylogenetic analysis was the Tamura-Nei (TN93)^61^ model, with the gamma distribution (G) and invariant sites (I), and it was estimated using MEGA version 7.0.26^60^. The phylogeny was estimated using the maximum likelihood (ML) method. The percentage of bootstrap values were obtained based on 1,000 repetitions.

Genetic divergences between sequences were estimated with the *p*-distance model applying the gamma distribution parameter using Mega version 7.0.26^60^. Descriptive statistics of genetic distances were done with the SPSS program version 25.0 for Windows (IBM SPSS Statistics, Armonk, NY: IBM Corp). A cut-off value for assigning new subtypes within genotype A was determined according to the *gag* fragment (399 bp). This was done by calculating the mean genetic distances among SRLV subtypes of A1–A20 (except for A6, A10, A14 and A15).

The deducted *gag-pol* amino acid sequences (214 aa) of 6 Iranian and 17 German *gag-pol* sequences were aligned with all available sequence data. The purposes of this alignment were: (1) Checking amino acid variations of epitopes 2 and 3, especially the N-terminus and C-terminus, as such substitutions may affect the sensitivity of ELISA. (2) Checking amino acid variations, with regard to epitopes 2 and 3, and other conserved motifs (GG and MHR), to be sure whether our sequence data have been genotyped correctly. Previous studies have shown some specific differences between different genotypes of A-C and E^9,15^. (3) Finding potential amino acid variation/s in our sequence data that presumably is linked to other lentiviruses of the family Retroviridae.

## Supplementary information

Supplementary Information.

## Data Availability

All data generated and analysed in this study are included in this published article and its supplementary information files.
