# Supplementary material for: Phylogenetic analysis of small ruminant lentiviruses in Germany and Iran suggests their expansion with domestic sheep
Source: Sci Rep. 2020 Feb 10;10:2243. doi: 10.1038/s41598-020-58990-9 (PMC7010740; doi:10.1038/s41598-020-58990-9)
Supplement: Supplementary file 1 — Supplementary Information. [file 41598_2020_58990_MOESM1_ESM.pdf]

Phylogenetic analysis of small ruminant lentiviruses in Germany and Iran suggests their expansion with domestic sheep

Vahid Molaee[1,*], Moira Bazzucchi[2], Gian Mario De Mia[2], Vahid Otarod[3], Darab Abdollahi[4], Sergio Rosati[5] & Gesine Lühken[1]

[1]Institute of Animal Breeding and Genetics, Justus Liebig University Giessen (JLU), Ludwigstraße 21, 35390 Gießen, Germany

[2]Istituto Zooprofilattico Sperimentale dell'Umbria e delle Marche Togo Rosati (IZSUM), Via G. Salvemini 1, 06126 Perugia, Italy

[3]Quarantine and Biosafety Directorate General, Iran Veterinary Organization (IVO), Vali Asr Avenue, Seyd Jamaledin Asad Abadi Street, 6349 Tehran, Iran

[4]Bureau of Animal Health and Disease Management, Iran Veterinary Organization (IVO), Vali Asr Avenue, Seyd Jamaledin Asad Abadi Street, 6349 Tehran, Iran

[5]Department of Veterinary Science, University of Turin (UNITO), Largo Paolo Braccini 2, 10095 Grugliasco, Torino, Italy

Supplementary Table S1. Estimates of evolutionary divergences of German and Iranian SRLV sequences compared to different subtypes of genotype B, C and E based on the *gag* fragment (nucleotide: 1114–1506; numbering according to prototype strain K1514[62]).

sequence	country	B1	B2	B3	mean B	C	E1	E2	mean E
MN233148	Germany	24.17	23.16	22.52	23.28	23.92	28.63	31.68	30.16
MN233105	Germany	23.16	22.52	22.01	22.56	23.16	27.23	28.5	27.87
MN233108	Germany	23.92	22.52	22.65	23.03	21.88	28.12	29.13	28.63
MN233107	Germany	22.90	22.26	20.99	22.05	23.16	26.72	28.24	27.48
MN233143	Germany	22.39	21.37	20.10	21.29	23.66	27.99	27.10	27.55
MN233124	Germany	21.63	21.63	19.97	21.08	24.43	26.59	26.84	26.72
MN233151	Germany	21.63	21.63	19.97	21.08	23.92	26.59	26.34	26.47
MN233104	Germany	23.54	19.47	20.99	21.33	20.87	25.70	27.35	26.53
MN233116	Germany	25.57	24.30	21.77	23.88	23.92	28.63	30.28	29.46
MN233111	Germany	23.54	24.68	21.25	23.16	24.68	28.63	30.28	29.46
MN233118	Germany	24.17	24.43	21.12	23.24	25.45	27.23	27.61	27.42
MN233132	Germany	25.19	25.70	21.63	24.17	25.95	26.59	29.39	27.99
MN233120	Germany	22.14	23.16	20.74	22.01	21.88	26.97	29.64	28.31
MN233145	Germany	23.92	22.39	21.50	22.60	22.65	26.59	28.63	27.61
MN233128	Germany	23.92	22.52	22.26	22.90	22.39	26.84	28.88	27.86
MN233141	Germany	24.30	24.68	22.26	23.75	22.90	28.12	30.41	29.27
MN233106	Germany	22.52	22.77	21.63	22.31	22.65	29.01	29.26	29.14
MK098477	Iran	25.45	24.43	21.50	23.79	24.68	27.23	28.88	28.06
MK098478	Iran	25.83	24.94	23.54	24.77	25.45	29.01	30.66	29.84
MK098479	Iran	23.92	22.90	20.10	22.31	22.65	27.35	30.28	28.82
MK098480	Iran	24.30	23.92	19.21	22.48	23.66	27.99	31.04	29.52
MK098481	Iran	25.19	25.19	21.37	23.92	23.66	25.83	29.90	27.87
MK098482	Iran	24.05	24.05	22.26	23.45	24.68	27.10	29.39	28.25

For each reference subtype up to two strains were selected and the evolutionary divergences calculated based on the mean divergence of each set of subtypes and representative sequences of this study. 17 German and six Iranian SRLV sequences were compared with subtypes/genotypes B1 (M33677 and GU120138), B2 (FJ195346 and EU010126), B3 (JF502417 and JF502416), C (AF322109), E1 (EU293537 and EF676025), and E2 (FR694921 and GQ381130).

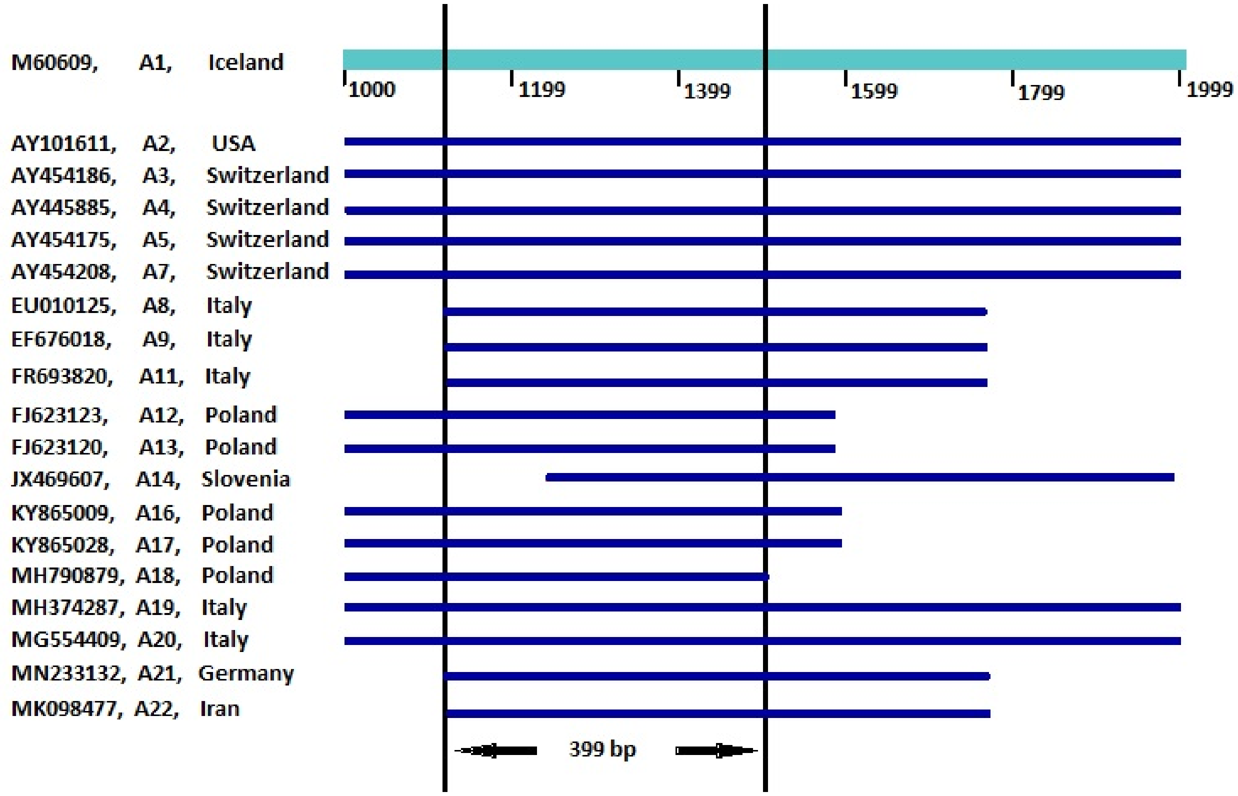

Supplementary Figure S1. Status of SRLV *gag-pol* fragment sequence data derived from previous studies and from the present study. The SRLV subtype A14 had to be excluded from analyses because of the shortness of the relevant sequence part. The sequence data available for the SRLV subtypes A12, A13, A16, A17 and A18 were not match with the sequence data of other SRLV subtypes of genotype A.

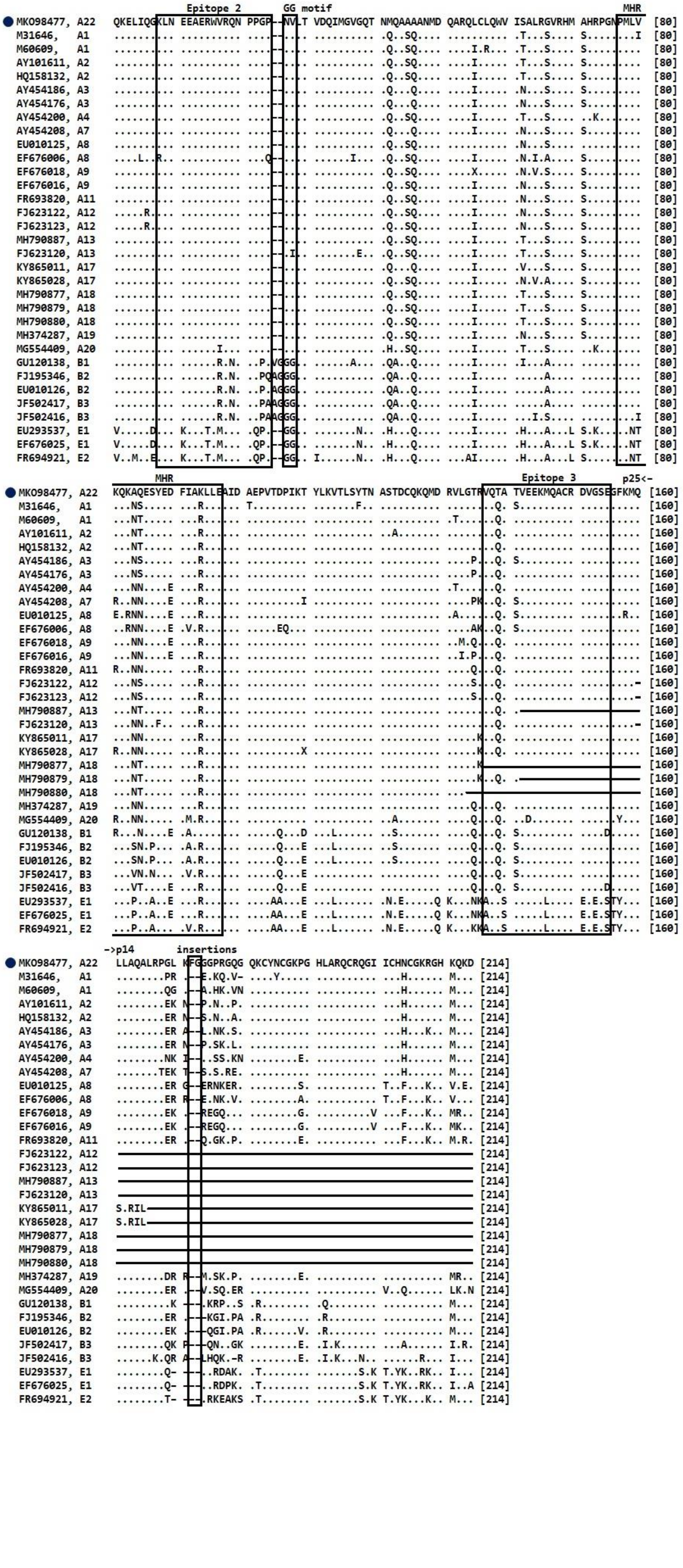

Epitope 2
GG motif
MHR
Epitope 3
p25<-
->p14
insertions

Supplementary Figure S2 related to Figure 2. Amino acid sequence alignments of the SRLV *gag-pol* fragment (not shown at the main text). The reference sequence is the SRLV sequence of strain BKH1 (accession number: MK098477) from the Iranian province of Chaharmahal-Va-Bakhtiari. Immunodominant epitopes 2 and 3, the major homology region (MHR), the double glycine motif (GG) and insertions (at positions 172 or 173) are delineated with boxes. The major core protein (p25) and nucleic acid-binding protein (p14) are separated with left and right arrows (p25 $\leq$ position160; p14 $\geq$ position161). Dashes and dots indicate deletions and identical residues, respectively.

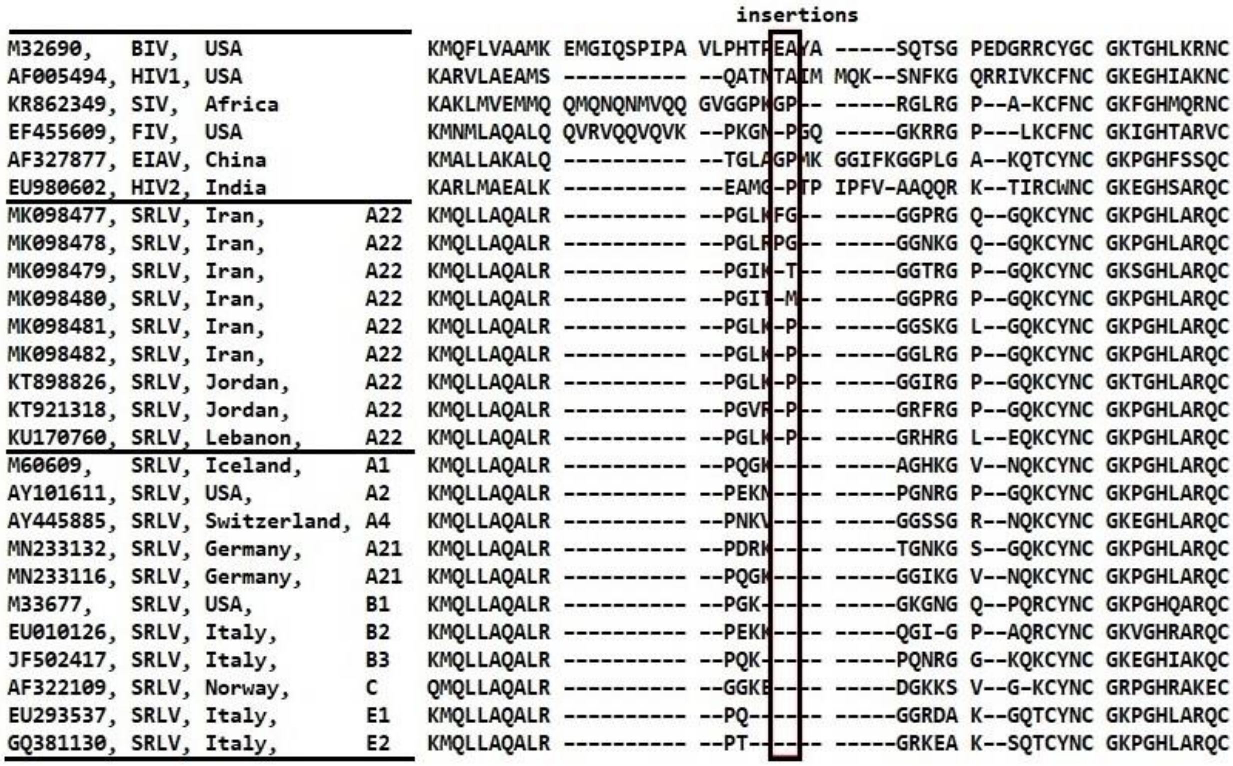

Supplementary Figure S3. Insertions (black box) on *gag-pol* fragment (amino acid at positions 172 or 173; numbering according to prototype strain BKH1, accession number: MK098477) were shown in different lentiviruses: bovine immunodeficiency virus (BIV), human immunodeficiency virus type 1 (HIV1), simian immunodeficiency virus (SIV), feline immunodeficiency virus (FIV), equine infectious anemia virus (EIAV), human immunodeficiency virus type 2 (HIV2) and small ruminant lentiviruses (SRLVs).
